# Contribution of Classic and Alternative Effector Pathways in Peanut-Induced Anaphylactic Responses

**DOI:** 10.1371/journal.pone.0028917

**Published:** 2011-12-14

**Authors:** Joost J. Smit, Karina Willemsen, Ine Hassing, Danielle Fiechter, Gert Storm, Louis van Bloois, Jeanette H. W. Leusen, Maarten Pennings, Dietmar Zaiss, Raymond H. H. Pieters

**Affiliations:** 1 Immunotoxicology, Institute for Risk Assessment Sciences, Utrecht University, Utrecht, The Netherlands; 2 Utrecht Centre for Food Allergy, Utrecht, The Netherlands; 3 Department of Pharmaceutics, Utrecht Institute for Pharmaceutical Sciences, Utrecht University, Utrecht, The Netherlands; 4 Utrecht University Medical Center, Utrecht, The Netherlands; 5 Faculty of Veterinary Sciences, Utrecht University, Utrecht, The Netherlands; Centre de Recherche Public de la Santé (CRP-Santé), Luxembourg

## Abstract

Food allergy affects approximately 5% of children and is the leading cause of hospitalization for anaphylactic reactions in westernized countries. However, the pathways of anaphylaxis in food allergy are still relatively unknown. We investigated the effector pathways of allergic and anaphylactic responses of different strains of mice in a clinical relevant model of peanut allergy. C3H/HeOuJ, C57BL/6 and BALB/c mice were sensitized by intragastric peanut extract and challenged by intragastric or intraperitoneal injection of peanut. Peanut-specific T cell responses, IgE, IgG1 and IgG2a and mucosal mast cell degranulation were induced to different extent in C3H/HeOuJ, C57BL/6 and BALB/c mice. Interestingly, anaphylactic symptoms after systemic challenge were highest in C3H/HeOuJ followed by C57BL/6 but were absent in BALB/c mice. Mechanistic studies showed that the food allergic systemic anaphylaxis was dependent on platelets, FcRγ and mast cells, and partially dependent on platelet activating factor and monocytes/macrophages, depending on mouse strain. These data demonstrate that in three mouse strains, components of the classic and alternative anaphylactic cascade are differently expressed, leading to differential outcomes in parameters of allergic disease and food induced systemic anaphylaxis.

## Introduction

Food allergy is generally defined as an adverse health effect arising from a specific immune response that occurs reproducibly on exposure to a given food. Although other food allergies such as milk allergy are generally outgrown in children, peanut allergy is persistent to adulthood [1], [2]. Even more concerning is that peanut and tree nut allergy account for more than half of all hospitalizations for anaphylaxis [3]. Despite that diagnosis of food allergy relies strongly on the detection of specific IgE and mast cell-mediated skin-prick testing, it is argued that allergic reactions may occur independently of antigen-specific IgE and mast cells [4].Already in the 70's it was demonstrated that human anaphylaxis could be mediated by IgG antibodies [5]. More recently, two pathways of systemic anaphylaxis have been demonstrated in mice: a classical pathway involving IgE, FcεRI, mast cells and histamine versus an alternative pathway mediated by IgG, FcγRIII, neutrophils, macrophages, basophils and platelet activating factor (PAF) [6], [7]. Interestingly, PAF was associated with the severity of anaphylaxis in humans [8], which was confirmed in a mouse study [9]. Nonetheless, most mouse studies looking at the role of the alternative pathway of anaphylaxis did not use relevant food allergens, or used mice preconditioned for responsiveness by using vasoactive mediators [10], [11], [12]. Therefore, until now no evidence exists for a role of alternative pathway in food allergy and food-induced anaphylactic responses.

Without doubt, animal models have contributed to the insight in the mechanisms of oral sensitization to food proteins. Generally, C3H/HeJ mice or C3H/HeOuJ mice are used [13], [14], [15], [16], [17], but other mouse strains, including BALB/c or C57BL/6 have been used in food allergy models as well [18], [19].

To assess the relevance of both pathways of anaphylaxis, we compared food allergic responses in C3H/HeOuJ, C57BL/6 and BALB/c mice. Marked differences were already observed in the level of oral sensitization to peanut, but most pronounced differences were observed with regard to anaphylactic responses in these mouse strains. These responses involved a variable, genetically determined combination of components of the classical and the alternative pathway of systemic anaphylaxis. This finding is of relevance to the human situation where inter-individual differences may be the cause of sometimes inconclusive diagnosis of and limited success of therapeutic approaches for food allergy.

## Results

### Specific antibody and T cell responses and mucosal mast cell degranulation differ considerably in different mouse strains

**Figure 1:**
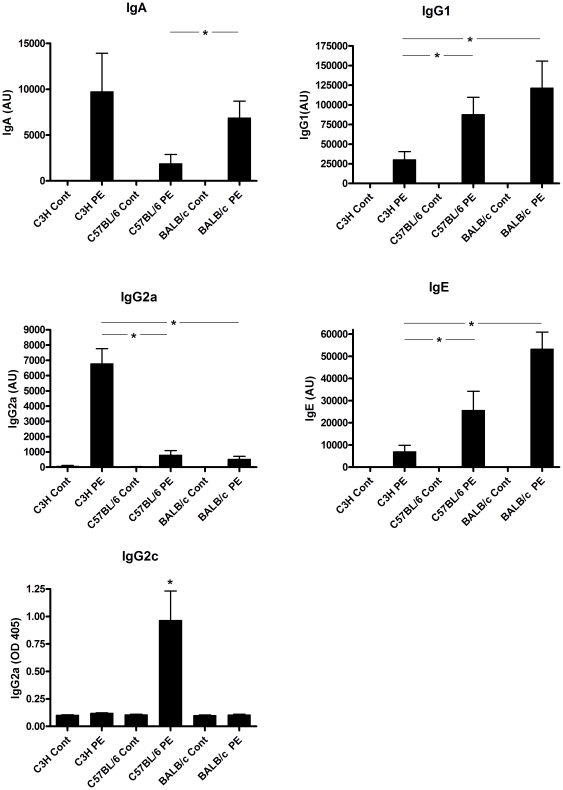
Antibody responses after PE oral sensitization in different mouse strains. C3H/HeOuJ (C3H), C57BL/6 and BALB/c mice were exposed to PBS+CT (cont) or PE+CT (PE) during a 4 week period, as described in material and methods. Graphs depict serum levels of PE-specific IgA, IgG1, IgG2a, IgE and IgG2c at day 36. Data are represented as mean AU± SEM of 8 mice. *: p<0.05 between indicated PE sensitized groups.

**Figure 2:**
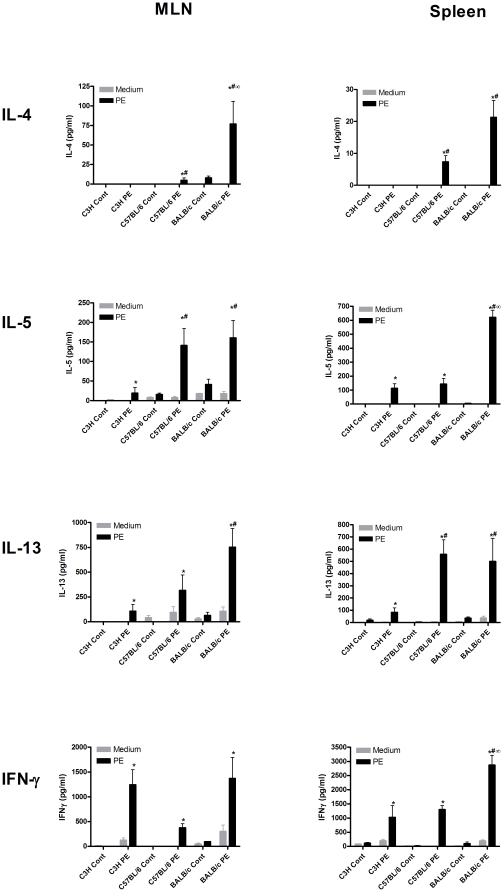
T cell responses after PE oral sensitization in different mouse strains. C3H/HeOuJ (C3H), C57BL/6 and BALB/c mice were exposed to PBS+CT (cont) or PE+CT (PE) during a 4 week period, as described in material and methods. Spleens and MLN were isolated and single cell suspensions of these tissues were incubated in the absence (medium) or presence of sterilized peanut extract (PE) in complete RPMI1640. Graphs depict levels of IL-4, IL-5, IL-13 and IFN-γ 96 h after stimulation. Data are represented as mean ± SEM of 6 mice. *: p<0.05 compared to control. #: p<0.05 compared to C3H/HeOuJ PE group. ∞: p<0.05 compared to C57BL/6 PE group.

First, we compared antibody responses in C3H/HeOuJ, C57BL/6 and BALB/c mice intragastrically exposed to peanut extract (PE) with cholera toxin (CT). Oral PE oral sensitization led to the induction of PE-specific IgA, IgG1, IgG2a and IgE in all mouse strains (figure 1). However, the levels of these antibodies differed significantly between these mouse strains. Levels of IgA and IgG2a were highest in C3H/HeOuJ mice while the levels of IgG1 and IgE were significantly higher in BALB/c mice. The small increase in IgG2a in C57BL/6 mice is likely due to cross reactivity of the used antibodies, since these mice do not express IgG2a but IgG2c instead (figure 1). To investigate the underlying T cell responses in the used mouse strains after oral sensitization, spleen and MLN cells were re-stimulated with PE (figure 2). This led to an induction of the Th2 cytokines IL-4, IL-5 and IL-13 in both spleen and MLN cells of C57BL/6 and BALB/c mice, but much less in C3H/HeOuJ mice. This induction was highest in BALB/c mice and significantly less pronounced in C3H/HeOuJ mice. IFN-γ was induced equally in all mouse strains in MLN cultures but was induced significantly higher in spleen cultures of BALB/c mice. This level of Th1 (IFNγ) cytokine induction after restimulation reflects a mixed *ex vivo* Th1/Th2 response in all strains in our model. Overall, oral sensitization and challenge with PE led to increased levels of PE-specific antibody and T cell responses, including IgE and Th2 cytokine production. Nonetheless, the level of these allergic parameters differed significantly among the used mouse strains.

### The degree of systemic anaphylaxis and ear swelling is mouse strain dependent

**Figure 3:**
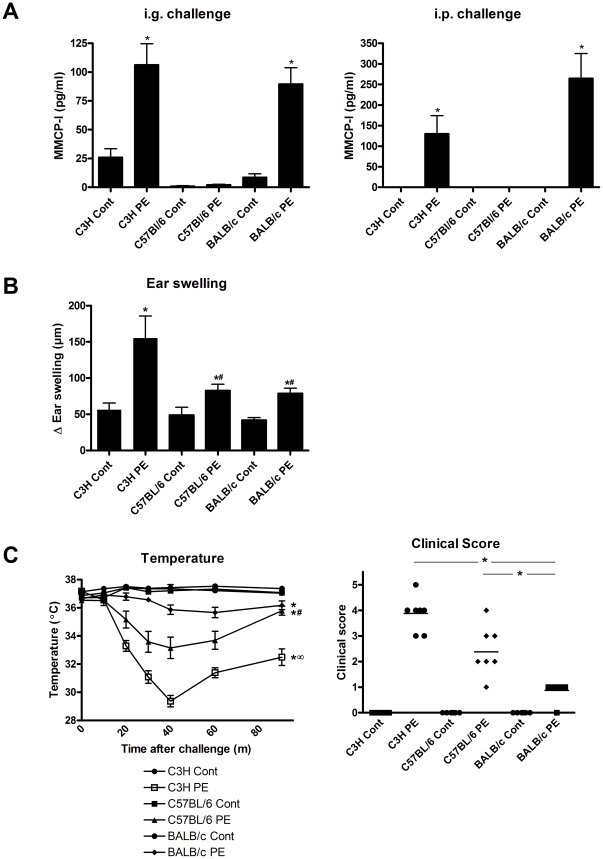
PE-induced mucosal mast cell degranulation, ear swelling and systemic anaphylaxis in different mouse strains. C3H/HeOuJ (C3H), C57BL/6 and BALB/c mice were exposed to PBS+CT (cont) or PE+CT (PE) during a 4 week period, as described in material and methods. **A**, Mice were challenged intragastrically (i.g.) or intraperitonially (i.p.) and blood was taken to measure MMCP-I. **B**, Mice were challenged intradermally with PE in the ear and ear swelling was measured after 3 hours. Ear swelling was calculated by correcting the thickness of the right ear with the left control ear thickness minus the basal ear thickness before challenge. **C**, Mice were challenged i.p. and rectal temperature was measured at indicated time points after challenge. In addition, peak anaphylactic scores were taken. Data are represented as mean ± SEM of 6–8 mice. *: p<0.05 compared to control. #: p<0.05 compared to BALB/c PE group. ∞: p<0.05 compared to BALB/c PE and C57BL/6 PE group.

Next, we assessed whether the differences in antibody and T cell responses in different mouse strains would lead to differences in clinical manifestations of food allergy as well. Therefore, we compared mast cell degranulation and systemic anaphylaxis after intra-gastric and systemic challenge with PE and observed that both challenges in PE-sensitized mice led to a significant release of, mucosal mast cell-derived, Mouse Mast Cell Protease-1 (MMCP-I) in the serum of C3H/HeOuJ and BALB/c, but not C57BL/6 mice (figure 3a). However, MMCP-I is not produced by connective tissue mast cells and to investigate the *in vivo* response of this type of mast cells, we measured mast cell-mediated ear swelling after intradermal injection of PE in the ears of sensitized mice. This showed that C3H/HeOuJ, C57BL/6 and BALB/c mice developed significant ear swelling upon challenge with PE (figure 3b). C3H/HeOuJ mice showed a significantly higher ear swelling compared to C57BL/6 and BALB/c mice.

Hereafter, we studied the development of systemic anaphylaxis after PE oral sensitization and systemic (i.p.) challenge. We measured an unambiguous anaphylactic response, reflected by a strong drop in temperature and increased clinical score, in C3H/HeOuJ and to lesser extent in C57BL/6 mice (figure 3c). Surprisingly, BALB/c mice only showed a very minimal anaphylactic response upon challenge. In addition to many previous attempts, oral challenge with PE of sensitized mice did not lead to any anaphylactic responses in all three mouse strains (data not shown).

Together, these data shows a clear discrepancy between mucosal and connective tissue mast cell degranulation and development of systemic anaphylaxis to PE in different mouse strains. C3H/HeOuJ mice displayed mucosal mast cell degranulation and the strongest level of systemic anaphylaxis. C57BL/6 mice did not show mucosal mast cell degranulation and exhibited less pronounced mast cell-mediated ear swelling and systemic anaphylaxis when compared to C3H/HeOuJ.

### PE-induced systemic anaphylaxis is FcRγ- mediated

**Figure 4:**
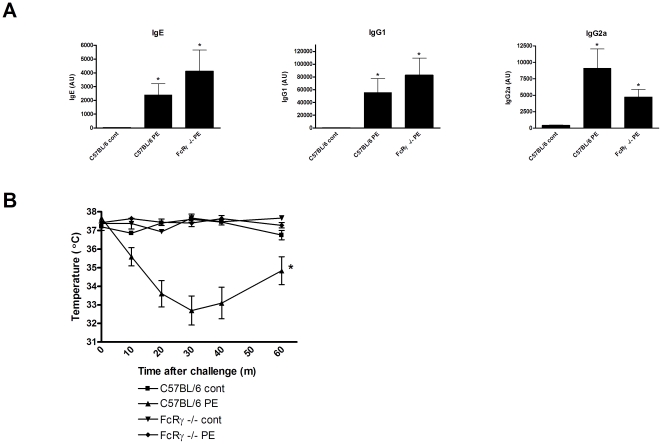
Humoral immunity and PE-induced systemic anaphylaxis. C57BL/6 and FcRγ −/− mice were exposed to PBS+CT (cont) or PE+CT (PE) during a 4 week period, as described in material and methods. **A**, Serum levels of PE-specific IgE, IgG1 and IgG2a at day 36. **B**, Temperature after i.p. challenge. Data are represented as mean ± SEM of 4–6 mice. *: p<0.05 compared to control.

Subsequently, to assess whether the observed PE-induced systemic anaphylaxis in C57BL/6 mice was IgG and IgE mediated, we used FcRγ−/− knockout C57BL/6 mice in our model. The Fc associated γ chain is a key component of FcγRI, III and IV and FcεRI but not of FcγRIIb and FcεRII (CD23). Therefore, in FcRγ−/− mice both IgG and IgE cannot bind with high affinity and/or activate responsive receptors. Remarkably, FcRγ−/− mice showed normal levels of IgE, IgG1 and IgG2a antibodies after oral sensitization in comparison to WT positive control mice (figure 4a), but did not develop systemic anaphylaxis upon PE challenge (figure 4b). This shows that PE-induced systemic anaphylaxis but not oral PE sensitization is FcRγ-mediated and dependent on activation via IgE or IgG.

### The role of basophils, neutrophils, monocytes/macrophages and mast cells in PE-induced systemic anaphylaxis

**Figure 5:**
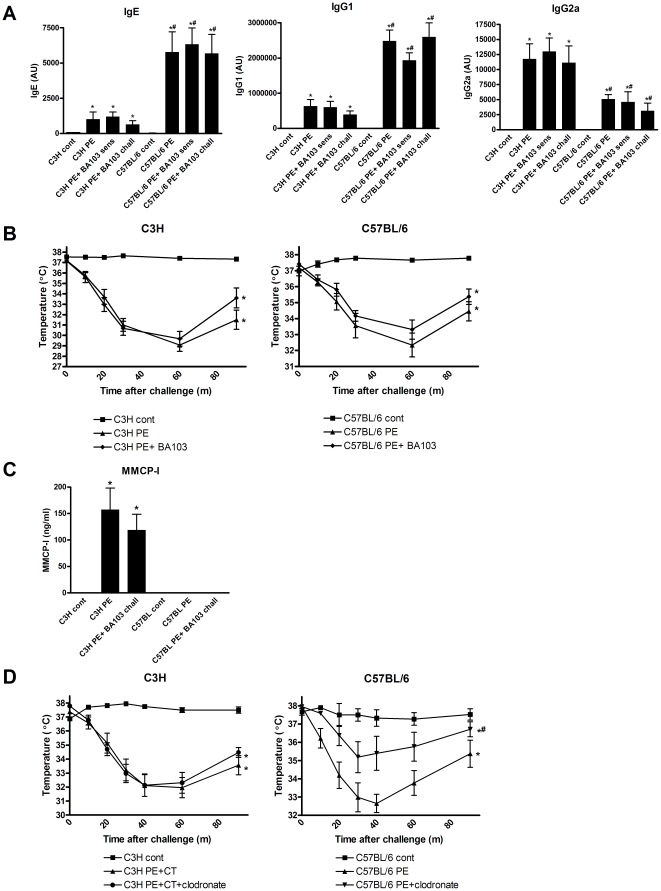
Basophils and monocytes/macrophages in PE-induced systemic anaphylaxis. C3H/HeOuJ (C3H) and C57BL/6 mice were exposed to PBS+CT (cont) or PE+CT (PE) during a 4 week period, as described in material and methods. Prior to oral sensitization (sens) or i.p. challenge (chall) indicated groups received BA103, a basophil depleting antibody. **A**, Serum levels of PE-specific IgE, IgG1 and IgG2a at day 36. **B**, Temperature after i.p. challenge. **C**, Serum MMCP-I levels after challenge. Prior to challenge, in indicated groups, monocytes/macrophages were depleted using clodronate liposomes. **D**, Temperature after i.p. challenge. Data are represented as mean ± SEM of 6–8 mice. *: p<0.05 compared to control. # p<0.05 compared to C3H PE group or to C57BL/6 PE group.

The occurrence of antibody mediated anaphylactic responses to PE despite no obvious mucosal mast cell response and a lower connective tissue mast cell response in C57BL/6 mice led us to explore alternative pathways of anaphylaxis in these mice compared to C3H/HeOuJ mice. It has been suggested that basophils, neutrophils or macrophages can contribute to or cause anaphylactic responses as well. Importantly, a comparison of the number of platelets, basophils, monocytes/macrophages and neutrophils showed no differences in the number of these cell types in spleen or peritoneal cavity of C3H/HeOuJ and C57BL/6 mice (figure S1). Therefore, we first investigated whether basophils influenced the development of systemic anaphylaxis in C57BL/6 mice and C3H/HeOuJ mice. Basophil depletion with the antibody BA103 did not influence PE oral sensitization as measured by levels of PE-specific IgG1, IgG2a and IgE (figure 5a) or systemic anaphylactic responses of C3H/HeOuJ and C57BL/6 mice (figure 5b). As a control, MMCP-I levels after PE challenge were equal among control and basophil-depleted mice (figure 5c), showing that mast cell responses were unaffected by BA103 treatment. In addition, neutrophil depletion using Gr-1 did not influence systemic anaphylactic responses of C3H/HeOuJ and C57BL/6 mice (figure S2). Hereafter, we investigated whether monocytes/macrophages play a role in PE-induced systemic anaphylaxis. Remarkably, selective depletion of monocytes/macrophages using clodronate liposomes did not affect the drop in temperature in C3H/HeOuJ mice, but it significantly lowered the anaphylactic response in C57BL/6 mice (figure 5d).

**Figure 6:**
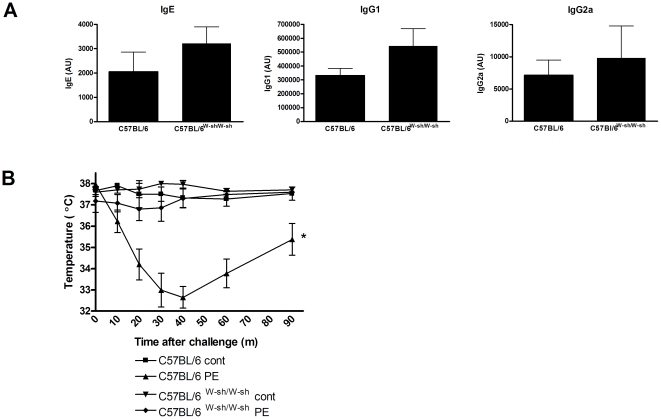
Mast cells in PE-induced systemic anaphylaxis. C57BL/6 WT and mast cell deficient C57BL/6^W-sh/W-sh^ mice were exposed to PBS+CT (cont) or PE+CT (PE) during a 4 week period, as described in material and methods. **A**, Serum levels of PE-specific IgE, IgG1 and IgG2a at day 36. **B**, Temperature after i.p. challenge. Data are represented as mean ± SEM of 6 mice. *: p<0.05 compared to control.

The induction of systemic anaphylaxis in the absence of mucosal mast cell degranulation was further examined in mast cell-deficient C57BL/6^W-sh/W-sh^ mice. Importantly, mast cell deficiency did not lead to differences in IgE, IgG1 or IgG2a levels compared to littermates (figure 6a). Interestingly, in contrast to C57BL/6 littermates C57BL/6^W-sh/W-sh^ mice did not develop any anaphylactic manifestations (figure 6b). Thus, basophils do not play a role in PE-induced oral sensitization and systemic anaphylaxis. In contrast, monocytes/macrophages do play a significant role in PE-induced systemic anaphylaxis in C57BL/6 mice, while playing no role in C3H/HeOuJ mice. This would suggest that parts of the alternative pathway of anaphylaxis, dependent on connective tissue mast cells, are mediating peanut-induced systemic anaphylaxis in C57BL/6 mice.

### PAF and platelets mediate PE-induced systemic anaphylaxis, depending on mouse strain

**Figure 7:**
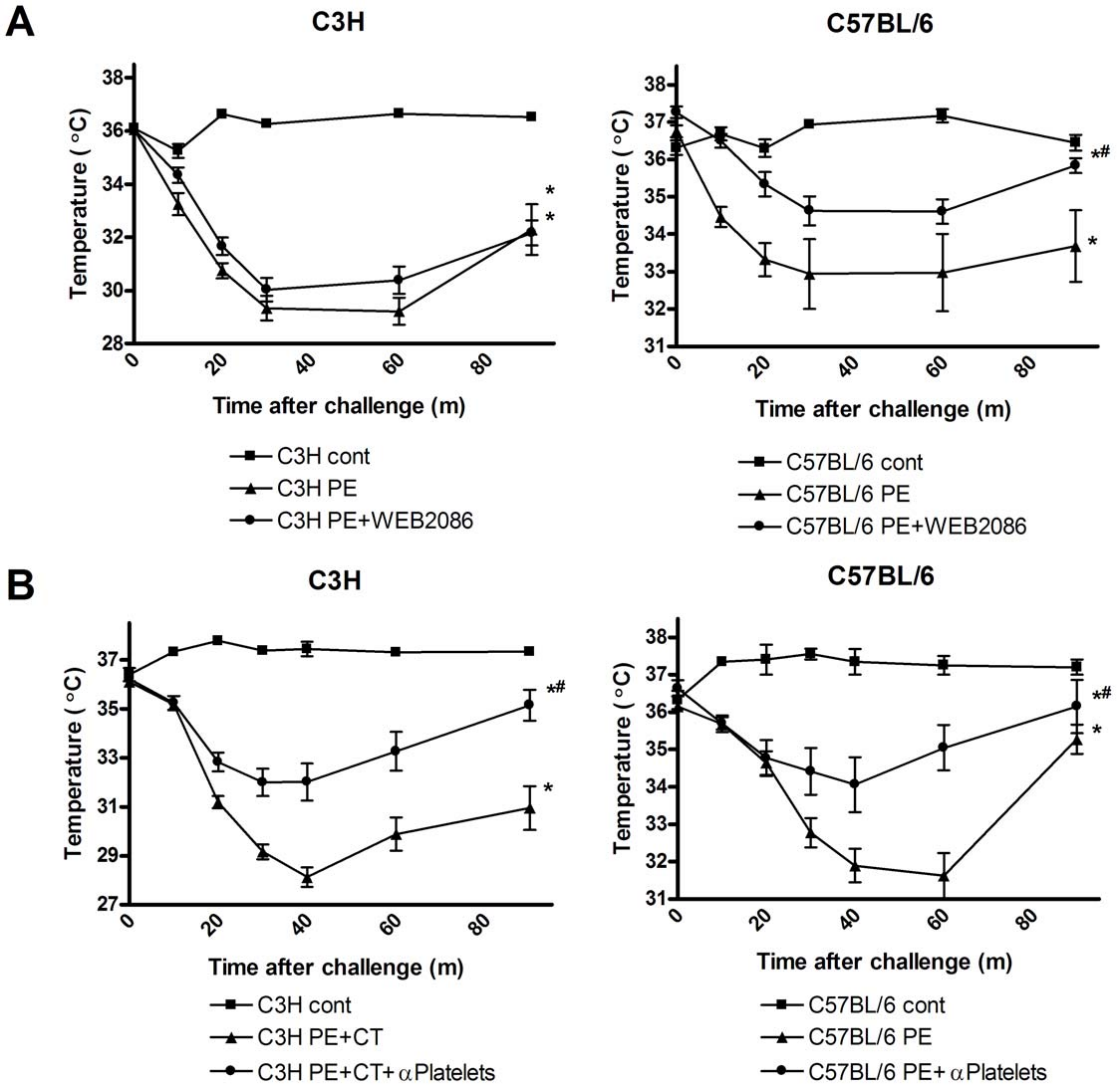
Platelet Activating Factor (PAF) and platelets in PE-induced systemic anaphylaxis. C3H/HeOuJ (C3H) and C57BL/6 mice were exposed to PBS+CT (cont) or PE+CT (PE) during a 4 week period, as described in material and methods. **A**, Temperature after i.p. challenge. Prior to challenge, indicated groups received the PAF R inhibitor WEB2086. **B**, Temperature after i.p. challenge. Prior to challenge, in indicated groups platelets were depleted using a rabbit anti-platelet serum. *: p<0.05 compared to control. # p<0.05 compared to PE groups.

PAF is considered an important mediator in the alternative pathway of anaphylaxis. For that reason, we looked whether inhibition of the PAF pathway would affect PE-induced systemic anaphylaxis. However, treatment before challenge with the PAFR inhibitor WEB2086 did not significantly inhibit systemic anaphylaxis responses in C3H/HeOuJ mice although the observed temperature drop did seem less severe at later time points after PAF inhibition (figure 7a). In contrast, PAF inhibition significantly lowered anaphylactic responses in C57BL/6 mice. Using another PAF R inhibitor, CV3988 gave the same results (data not shown). Antibody-mediated depletion of platelets did significantly inhibit systemic anaphylaxis in both C3H/HeOuJ and C57BL/6 mice (figure 7b). Therefore, although PE induced systemic anaphylaxis involves platelets in both C3H/HeOuJ and C57BL/6 it only significantly involves PAF in C57BL/6 mice. Again suggesting that parts of the alternative pathway of anaphylaxis are mediating peanut-induced systemic anaphylaxis in C57BL/6 mice

## Discussion

Manifestations of food allergy and food induced anaphylaxis may vary widely among individuals, even among patients reacting to the same responsible allergen. In addition, the underlying immunological mechanisms of these differences may be the reason why a classical diagnosis of food allergy and/or anaphylaxis is not always conclusive. In an attempt to reveal possible mechanisms of this pleiomorphy, we set out to investigate and compare parameters and manifestations of food allergy in different well-known mouse strains, i.e. C3H/HeOuJ, C57BL/6 and BALB/c mice. We observed that oral sensitization to PE, when comparing IgE and Th2 cytokine levels, was most effective in BALB/c mice, followed by C57BL/6 mice, and least effective in C3H/HeOuJ mice. Remarkably, the degree of systemic anaphylaxis was strain-dependent but, in contrast to oral sensitization, consistently most severe in C3H/HeOuJ, less pronounced in C57BL/6 and very low or minimal in BALB/c mice. This observed differences obviously point to variable genetic susceptibility to food sensitization in mice, as in human subjects.

The parameters of food sensitization in BALB/c mice in our model were different from some observations in literature, reporting lower levels of IgE and Th2 responses in these mice [14], [20]. Notably, anaphylaxis in BALB/c mice is clearly allergen-dependent as evidenced by the findings that less complex allergens such as ovalbumin or ß-lactoglobuline [11], [18] induce anaphylactic responses in these mice. Furthermore, the challenge dose and route of exposure is very important in this model. We as well as others [19] were not able to observe anaphylactic responses after various intragastric doses of PE in any mouse strain. But orally induced anaphylaxis could be demonstrated to very high amounts of protein (200 mg protein/mouse), comparable to 700 grams or more than 1000 peanuts in a human situation [17]. The occurrence of anaphylaxis after either systemic exposure, or after extreme oral doses or to single purified and relatively small allergens is in line with a recent study showing that ingested allergens must be absorbed systemically to induce anaphylaxis [21]. Notwithstanding, the anaphylactic responses measured in our studies after oral sensitization and systemic challenge are strong and consistent, resulting in a robust model and a notable experimental advantage. In this model, BALB/c mice did not show anaphylaxis despite a relative high level of expression of Th2 cytokines and IgE after oral sensitization. In addition, despite high levels of IgE and the occurrence of systemic anaphylaxis, C57BL/6 mice did not show increased mucosal mast cell degranulation upon intra-gastric or systemic PE challenge. This clearly points to multiple mechanisms involved in susceptibility to peanut-induced anaphylaxis.

Classically, the mast cell has always been the central mediator of allergy-mediated anaphylaxis. Two types of mast cells have been described in the mouse: mucosal mast cells expressing MMCP-I and connective tissue mast cells [22], [23], [24]. To investigate the responses of connective tissue mast cells, we measured acute ear swelling, which occurred in C57BL/6 mice and in BALB/c mice but clearly less than C3H/HeOuJ mice. This suggests that the observed differences in systemic anaphylactic symptoms between the mouse strains may be due to differential involvement of either mucosal or connective tissue mast cell subsets. In addition, the apparent lack of involvement of mucosal mast cells in C57BL/6 led us to investigate the role of the classical versus alternative pathways of anaphylaxis in these mice compared to C3H/HeOuJ mice.

The role of the alternative pathway of anaphylaxis involving IgG, macrophages, basophils and PAF in food allergy and food-induced anaphylaxis has already been suggested [6]. Recently, studies by Jönsson suggested a novel alternative pathway in which neutrophils, activated via IgG contributed to systemic anaphylactic shock in mice as well [7].In our experiments, peanut-induced systemic anaphylaxis in C57BL/6 mice was shown to be entirely dependent on FcRγ and mast cells and partially dependent on PAF, platelets and monocytes/macrophages. Systemic anaphylaxis in C3H/HeOuJ was also dependent in platelets, but not on PAF or monocytes/macrophages. Despite earlier reports on the role of basophils in other allergic diseases [25], basophils did not play a role in oral sensitization and systemic anaphylaxis in any of the strains. Moreover, neutrophil depletion using Gr-1 did not influence systemic anaphylactic responses of C3H/HeOuJ and C57BL/6 mice in our studies. This suggests that the involvement of components of the alternative anaphylaxis pathway, i.e. macrophages and PAF, in food allergy is strain-dependent but still dependent on mast cell activation.

The earlier mentioned studies investigating alternative pathways were performed in mouse models using passive cutaneous or systemic anaphylaxis, penicillin-, or TNP-OVA-mediated anaphylaxis [6], [7], [11], [12]. Moreover, these experiments were done with substantial immunization protocols (such as Freund's adjuvant) followed by injection of large amounts of antigen, which may not recapitulate the development of hypersensitivity in food allergy. More relevant to food allergy, it has been shown that in mice preconditioned for responsiveness to vasoactive mediators, peanuts could directly induce anaphylaxis by an IgE-independent but alternative pathway-dependent mechanism [10]. In addition, in a classical mouse model of food allergy using C57BL/6, Arias et al showed that a PAF-antagonist, but not a histamine antagonist inhibited systemic PE anaphylaxis [9].Recently in the same model, very high amounts (3.75 mg) of peanut injected intravenously induced anaphylaxis in mast cell deficient mice, which was dependent on macrophages and to lesser extent on basophils [26]. Again, this shows that probably the dose and route of exposure of allergen is crucial in which pathway of anaphylaxis will be involved. A very high dose or systemic exposure of allergen may involve the alternative pathway of anaphylaxis, as suggested by others [11].

It has been demonstrated that PAF was an important mediator of systemic anaphylaxis in the earlier mentioned models [9], [27]. We now append these studies showing that the role of PAF, and additionally of monocytes/macrophages is strain-dependent. PAF is produced by monocytes/macrophages, mast cells and many other immune cells, activates platelets but also has effects on vascular and endothelial cells, explaining its possible role in systemic anaphylaxis [28]. A human study showed increased levels of PAF during anaphylaxis and more severe anaphylaxis in individuals who slowly catabolize PAF [8]. Moreover, it was suggested that PAF secretion by monocytes/macrophages, via platelets, contributes to pathophysiology of allergic asthma [29]. We therefore can envision a situation in food-induced anaphylaxis where initial activation of mast cells and secretion of histamine and PAF is followed by activation of monocytes/macrophages leading to secretion of additional PAF and activation of platelets. However, the present findings suggest that this may be determined by predisposing genetically factors in man as it is in the mouse. We consider it very well possible that the relatively high level of mast cell degranulation in C3H/HeOuJ mice and humans displaying the classical pathway obscures a possible activation and involvement of components of the alternative pathway.

Previous mechanistic studies indicate that the present mouse model of peanut allergy resembles the situation in human patients to a great extent. Classically, in humans, food allergy and food-induced anaphylaxis is suggested to be mostly IgE mediated [6]. However, the current study showing strain-dependent differences in the role of classical versus alternative pathways may be translational to the human situation. Some patients may also display alternative pathway-driven clinical effects (e.g. PAF expression) and thus resemble a genetically predisposed mouse strain as the C57BL/6 mouse. Our studies are in line with the suggestion by Finkelman that the differences between mouse and man in this respect may be caused by the used mouse models more than by a difference in immune system between mouse and human [6].

In summary, in this study we describe marked differences in parameters and manifestations of food allergy in different mouse strains. In addition, we show a role of components of the alternative pathway of anaphylaxis in a food allergy model. However, clear differences exist between mouse strains in participation of this pathway. In conclusion, clinical manifestations of food allergy and anaphylaxis are not simply and directly linked to the classical components of the allergic cascade. This might explain the pleiomorphic manifestations of the clinical phenomena in the human situation. In addition, these findings may have important implications for diagnosis of and finding new targets for treatment of food allergy and food-induced anaphylaxis.

## Methods

### Mice

Five-week-old specific pathogen-free female C3H/HeOuJ, C57BL/6J, and BALB/cByJ mice were purchased from Charles River (France) and housed under specific pathogen-free conditions within the animal care facility at the Utrecht University. Mast cell-deficient C57BL/6^W-sh/W-sh^ or FcRγ−/− mice, both on a C57BL/6 background, were bred and maintained at the animal care facility at the Utrecht University. Experiments in this study were approved by the Animal Experiments Committee of the Utrecht University (ID #2011.III.03.031).

### Peanut oral sensitization and challenge

Peanuts were kindly provided by Intersnack BV (the Netherlands) and peanut extract (PE) was prepared as previously described [15]. Cholera toxin (CT) was obtained from List Biological Laboratories, Inc. (CA, USA) To elicit oral sensitization to PE, mice were intragastrically (i.g.) dosed with 6 mg PE plus 15 µg CT per mouse for three consecutive days, and this was repeated every week for four weeks (exposure on days 0, 1, 2, 7, 14, 21 and 28). Control groups received PBS plus 15 µg CT. Thereafter, mice received an i.g. challenge of 15 mg PE on day 35 and were sacrificed one day later.

### Measurement of ear swelling

Acute ear swelling in PE-sensitized mice was determined after intradermal challenge with 10 µg PE in the right ear pinnae. As a negative control, mice were challenged with PBS in the left ear. Before challenge and after 3 hours, ear thickness of both ears was measured in duplicate using a digital micrometer (Mitutoyo, the Netherlands). Ear swelling was calculated by correcting the thickness of the right ear with the left control ear minus the basal ear thickness before challenge.

### Measurement of systemic anaphylaxis

In separate experiments, mice received a challenge of 1 mg PE intraperitoneally (i.p.) on day 35. As an objective parameter of anaphylactic shock, body temperature was measured by means of rectal thermometry every 10–20 minutes for 90 minutes after challenge. In addition, clinical symptoms were scored using a scoring system, as used before [19].

### Cell and cell mediator antagonists

PAF function was inhibited using the PAF-receptor antagonists WEB 2086 or CV 3988 (Biomol, USA), which were injected i.p. one hour before challenge at 5 mg/kg BW. Mouse blood platelets were depleted using a rabbit anti mouse platelet serum (Accurate Chemical & Scientific corp, USA) at 50 µl/mouse i.p. three hours before challenge [30]. Depletion of platelets was confirmed by whole blood analysis (Abbott Cell-Dyn CD-1800 Hematology Analyzer, USA) (figure S1). Basophils were depleted with one i.p. injection of 50 µg BA103, one day before oral sensitization or challenge as described previously [31] (figure S1). Monocytes/macrophages were depleted 3 days before challenge using liposomes containing dicloromethylene-biphosphonate (clodronate), as described previously [32] at 5 mg/mouse i.p. Macrophage depletion was confirmed by differential cell counts and flowcytometric analysis of F4/80 + cells in peritoneal fluid (figure S1). In addition, clodronate liposome treatment did not affect FcεRI ^+^ cells (mast cells/basophils) in peritoneal fluid. Neutrophils were depleted one day before challenge by i.p. injection of 500 µg/mouse Gr-1 antibody [33].

### Measurement of PE-specific antibodies and MMCP-I in serum

PE-specific IgG1, IgG2a, IgG2c and IgE in serum of day 36 were measured by ELISA as described previously [34]. This protocol was amended: a positive pool serum derived from PE/alum sensitized mice was used as reference value to calculate Arbitrary Units (AU). Levels of IgG2c were depicted as OD at 405 nm. In addition, serum was collected within 45 minutes after oral challenge and 3 hours after i.p. challenge on day 35, and MMCP-I was determined using a specific ELISA kit according to instructions of the manufacturer (Moredun Scientific, Scotland).

### Cell culture and cytokine measurement

Spleen and MLN single cell suspensions (2.5×10^6^/ml) were incubated in the presence or absence of PE (200 µg/ml) for 96 h at 37°C in complete RPMI1640 with 10% FCS, after which culture supernatants were harvested and stored at −20°C until analysis, as described before [14]. Levels of IFN-γ, IL-4, IL-5 and IL-13 in culture supernatants were determined by sandwich ELISA according to the instructions of the manufacturers (eBioscience, Austria).

### Statistical analysis

Data are presented as means ± standard error of the mean (SEM) and analyzed using GraphPad Prism software. Antibody, MMCP-I and cytokine levels and data on ear swelling were logarithmic transformed followed by a one-way ANOVA and Bonferroni as a post-hoc test. Temperature curves were statistically analyzed using a repeated measures ANOVA and clinical scores were statistically analyzed by the Kruskall-Wallis test.

## Supporting Information

Figure S1 **Number of platelets, basophils, macrophages and neutrophils in control and depleted mice.** (**A**) Number of platelets in blood of control and mice treated with a rabbit anti-mouse platelet serum. The number of platelets was performed by whole blood analysis on a hematology analyzer. Data are represented as mean ± SEM of 4 mice. # p<0.001 compared to untreated C3H or C57BL/6 mice. (**B**) Number of basophils in spleens of control and mice treated with BA103, a basophil depetion antibody. Cells were analyzed using flow cytometry, gated based on FSC-SSC pattern and FcεRα+ and CD49b+ staining for basophils. Pictures show representative dot plots with indicated average number of gated cells. (**C**) Number of macrophages in peritoneal fluid of control and mice treated with monocyte/macrophage depleting clodronate liposomes. Cells were analyzed using flow cytometry, gated based on FSC-SSC pattern and GR-1-CD11b+F4/80+ staining for macrophages. Pictures show representative dot plots with indicated average number of gated cells. (**D**) Number of macrophages in peritoneal fluid of control and mice treated with monocyte/macrophage depleting clodronate liposomes. Cytospins were made of peritoneal washings and stained with DiffQuick (H&E). Pictures show representative micrographs at 20× magnification. (**E**) Number of neutrophils in spleen of control and mice treated with anti-Gr-1. Cells were analyzed using flow cytometry, gated based on FSC-SSC pattern and GR-1+CD11b+F4/80− staining for neutrophils. Pictures show representative dot plots with indicated average number of gated cells.
(TIF)

Figure S2 **Neutrophils in PE-induced systemic anaphylaxis.** C3H/HeOuJ (C3H) and C57BL/6 mice were exposed to PBS+CT (cont) or PE+CT (PE) during a 4 week period, as described in material and methods. Prior to i.p. challenge indicated groups received Gr-1, a neutrophil depleting antibody. Depicted is the temperature after i.p. challenge. Data are represented as mean ± SEM of 6–8 mice. *: p<0.05 compared to control.
(TIF)
