# Supplementary figures and images for: Contribution of Classic and Alternative Effector Pathways in Peanut-Induced Anaphylactic Responses
Source: PLoS One. 2011 Dec 14;6(12):e28917. doi: 10.1371/journal.pone.0028917 (PMC3237567; doi:10.1371/journal.pone.0028917)

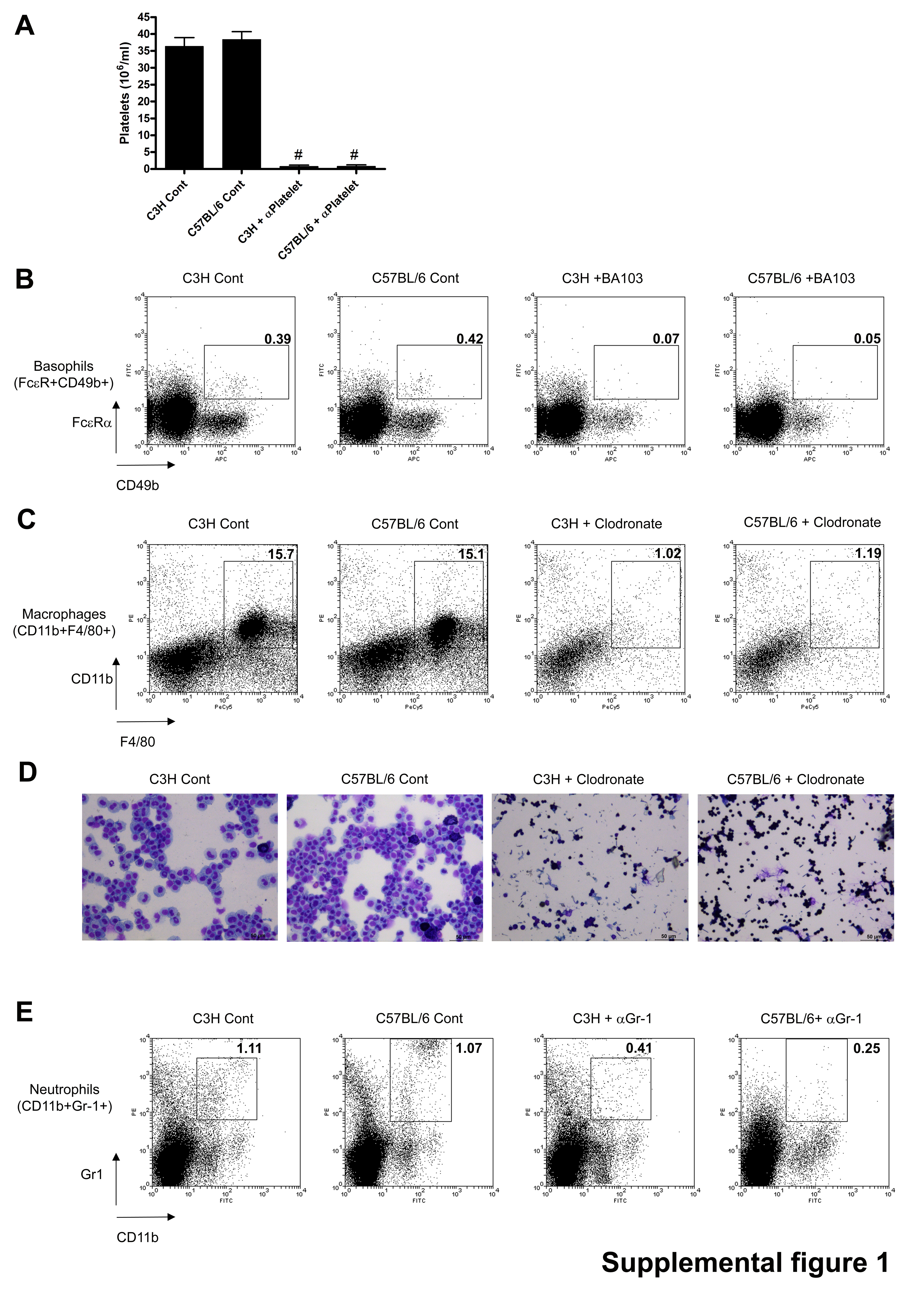

Supplement: Figure S1 — Number of platelets, basophils, macrophages and neutrophils in control and depleted mice. (A) Number of platelets in blood of control and mice treated with a rabbit anti-mouse platelet serum. The number of platelets was performed by whole blood analysis on a hematology analyzer. Data are represented as mean ± SEM of 4 mice. # p<0.001 compared to untreated C3H or C57BL/6 mice. (B) Number of basophils in spleens of control and mice treated with BA103, a basophil depetion antibody. Cells were analyzed using flow cytometry, gated based on FSC-SSC pattern and FcεRα+ and CD49b+ staining for basophils. Pictures show representative dot plots with indicated average number of gated cells. (C) Number of macrophages in peritoneal fluid of control and mice treated with monocyte/macrophage depleting clodronate liposomes. Cells were analyzed using flow cytometry, gated based on FSC-SSC pattern and GR-1-CD11b+F4/80+ staining for macrophages. Pictures show representative dot plots with indicated average number of gated cells. (D) Number of macrophages in peritoneal fluid of control and mice treated with monocyte/macrophage depleting clodronate liposomes. Cytospins were made of peritoneal washings and stained with DiffQuick (H&E). Pictures show representative micrographs at 20× magnification. (E) Number of neutrophils in spleen of control and mice treated with anti-Gr-1. Cells were analyzed using flow cytometry, gated based on FSC-SSC pattern and GR-1+CD11b+F4/80− staining for neutrophils. Pictures show representative dot plots with indicated average number of gated cells. (TIF) [file pone.0028917.s001.tif]
